# Comparison between next‐generation sequencing and multiplex polymerase chain reaction assays for nonsmall‐cell lung cancer molecular diagnosis

**DOI:** 10.1002/cam4.7162

**Published:** 2024-04-04

**Authors:** Shuji Murakami, Kanako Shinada, Yuka Otsutsumi, Fumiko Komine, Yuan Yuan, Junko Nakamura, Seigo Katakura, Tetsuro Kondo, Terufumi Kato, Tomoyuki Yokose, Haruhiro Saito

**Affiliations:** ^1^ Department of Thoracic Oncology Kanagawa Cancer Center Yokohama Kanagawa Japan; ^2^ Department of Pathology Kanagawa Cancer Center Yokohama Kanagawa Japan; ^3^ Riken Genesis Co., Ltd. Shinagawa‐ku Tokyo Japan

**Keywords:** AmoyDx pan lung cancer PCR panel, nonsmall‐cell lung cancer, Oncomine Dx Target Test

## Abstract

**Purpose:**

Genetic mutation detection has become an important step in nonsmall‐cell lung cancer (NSCLC) treatment because of the increasing number of drugs that target genomic rearrangements. A multiplex test that can detect multiple gene mutations prior to treatment is thus necessary. Currently, either next‐generation sequencing (NGS)‐based or polymerase chain reaction (PCR)‐based tests are used. We evaluated the performance of the Oncomine Dx Target Test (ODxTT), an NGS‐based multiplex biomarker panel test, and the AmoyDx Pan Lung Cancer PCR Panel (AmoyDx PLC panel), a real‐time PCR‐based multiplex biomarker panel test.

**Materials and Methods:**

Patients with histologically diagnosed NSCLC and a sufficient sample volume to simultaneously perform the AmoyDx PLC panel and ODxTT‐M were included in the study. The success and detection rates of both tests were evaluated.

**Results:**

Biopsies revealed 116 cases of malignancies, 100 of which were NSCLC. Of these, 59 met the inclusion criteria and were eligible for analysis. The success rates were 100% and 98% for AmoyDx PLC panel and ODxTT‐M, respectively. Nine driver mutations were detected in 35.9% and 37.3% of AmoyDx PLC and ODxTT‐M panels, respectively. *EGFR* mutations were detected in 14% and 12% of samples using the AmoyDx PLC panel and ODxTT‐M, respectively. Of the 58 cases in which both NGS and AmoyDx PLC panels were successful, discordant results were observed in seven cases. These differences were mainly due to different sensitivities of the detection methods used and the gene variants targeted in each test.

**Discussion:**

The AmoyDx PLC panel, a PCR‐based multiplex diagnostic test, exhibits a high success rate. The frequency of the nine genes targeted for treatment detected by the AmoyDx PLC panel was comparable to the frequency of mutations detected by ODxTT‐M. Clinicians should understand and use the AmoyDx PLC panel and ODxTT‐M with respect to their respective performances and limitations.

## Graphical abstract

The analysis, which included 59 NSCLC cases, found both tests to have high success rates (AmoyDx PLC 100%, ODxTT 98%) and similar mutation detection rates (around 36%). Discordant findings in seven cases were attributed to differences in sensitivity and gene variants targeted by each test. The AmoyDx PLC panel is a reliable PCR‐based test for NSCLC mutation detection, and clinicians should consider the performance and limitations of both testing methods.

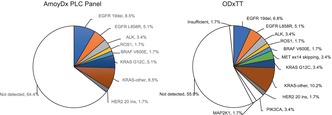

## 1 INTRODUCTION

Molecularly targeted drugs have been developed for various oncogenic driver mutations in advanced nonsmall‐cell lung cancer (NSCLC).^1^ These drugs dramatically improve the survival of patients with advanced NSCLC.^1^ Therefore, molecular testing prior to treatment initiation for advanced NSCLC is essential. Currently, the main nine driver mutations targeted in advanced NSCLC are the following: epidermal growth factor receptor (*EGFR*)‐mutations, anaplastic lymphoma kinase (*ALK*)‐fusions, ROS1 proto‐oncogene (*ROS1*)‐fusions, *KRAS* mutations, *BRAF* V600E mutation, mesenchymal‐epithelial transition (*MET*) exon 14 skipping, *RET* fusions, human epidermal growth factor receptor 2 (*HER2*) mutations, and neurotrophic tyrosine receptor kinase (*NTRK*) fusions.

As the number of testable genetic mutations increases, multiplex diagnostic systems that can simultaneously test for multiple genes are becoming increasingly relevant.^2^ One such system is the next‐generation sequencing (NGS)‐based Oncomine DX Multi‐Target Test system (ODxTT‐M; Thermo Fisher Scientific, Waltham, MA, USA). ODxTT‐M simultaneously evaluates 46 cancer‐related genes (supplementary Table 1, supplementary Table 2), including the nine therapeutic target genes, and is the first NGS panel for NSCLC testing that was granted reimbursement coverage in Japan in June 2019.^3^

ODxTT‐M has been widely applied in daily clinical practice in Japan; however, it has several limitations.^4^ ODxTT‐M requires specimens that contain nucleic acids of sufficient quality and quantity.^5^ Poor quality or too few tumor samples can cause ODxTT‐M analysis failure.^5^, ^6^ Consequently, the estimated tumor content of the biopsy sample is recommended to be ≥30% of the total cells. Furthermore, a sufficient amount of tissue is required to enable successfully NGS technology using ODxTT‐M.^5^, ^6^

The AmoyDx Pan Lung CancerPCR Panel (AmoyDx PLC panel; Amoy Diagnostics Co., Ltd., Xiamen, China), a real‐time PCR‐based multiplex diagnostic test, was granted reimbursement coverage in Japan in January 2022. AmoyDx PLC panel specifically detects genetic mutations in lung cancer, covering nine driver mutations that are targets of molecular‐targeted drugs (Tables S1 and S3). Real‐time PCR is expected to reduce testing time because it requires fewer testing steps than NGS, and the AmoyDx PLC panel is expected to enable analysis of smaller amounts of tissue samples, which will increase the success rate of testing, thus enabling the molecular testing of more patients.

We previously compared the performance of ODxTT‐M and the Cobas EGFR Mutation Test v2 (Cobas EGFR: Roche Molecular Systems) in detecting *EGFR* mutations and reported a higher test success rate for Cobas EGFR and a high concordance except for a few discrepancies between the two tests.^7^ In this study, we evaluated the performance of ODxTT‐M, an NGS‐based multiplex biomarker panel, and the AmoyDx PLC panel, a real‐time PCR‐based multiplex biomarker panel in the detection of nine driver mutations.

## 2 PATIENTS AND METHODS

### 2.1 Aim and study design

This study aimed to evaluate the accuracy of the AmoyDx PLC panel in biopsy specimens while simultaneously analyzing ODxTT‐M using the same tumor biopsy specimens that met the criteria for ODxTT‐M test submission. In this single‐center prospective study, the success rate of the biopsy‐based AmoyDx PLC panel tests was evaluated as the primary endpoint. Secondary endpoints were as follows: (1) mutation call profile using the AmoyDx PLC panel and concordance between ODxTT‐M and the AmoyDx PLC panel in detecting nine lung cancer‐related genes (*EGFR*, *BRAF*, *ALK*, *ROS1*, *MET*, *RET*, *KRAS*, *HER2*, and *NTRK*); (2) discordant case validation between ODxTT‐M and the AmoyDx PLC panel; (3) comparison of the amounts of extracted nucleic acids (DNA/RNA) measured using fluorescence and absorbance. Because the success rate of previous ODxTT‐M tests at our institution was approximately 95% (assuming a threshold success rate of 95%), an expected success rate of 100%, an alpha error of 0.05 per side, and a beta error of 0.8 per side, indicated that the minimum number of patients required would be 59; therefore, the number of patients enrolled would be 60, assuming ineligible cases. Consent acquisition was terminated when the number of analyzed cases reached 60. Written informed consent was obtained from all patients who were diagnosed with lung cancer and scheduled for biopsy. This study was conducted in accordance with the Declaration of Helsinki and approved by the Institutional Review Board of Kanagawa Cancer Center Hospital, Japan (2021EKI‐148).

### 2.2 Patient selection

Study subjects were individuals aged 20 years or older with clinically suspected lung cancer and in need of histological biopsy examination, who provided written consent to participate in the study. Finally, written informed consent was obtained from 148 patients who were scheduled to undergo biopsy examination between April and October 2022 at the Kanagawa Cancer Center Central Hospital. Among them, the analytes were histologically NSCLC and had sufficient tumor tissue to be submitted to the AmoyDx PLC panel and ODxTT‐M. In these cases, if the specimen was histologically diagnosed as NSCLC and the pathologist determined that there was sufficient tissue volume, the patient was enrolled in the study, and biomarker analysis was performed simultaneously using the AmoyDx PLC panel and ODxTT‐M.

### 2.3 Tissue sample acquisition

Tissue sampling procedures included transbronchial lung biopsy (TBLB) using endobronchial ultrasonography with a guide sheath (EBUS‐GS), endobronchial biopsy (EBB) using direct‐vision forceps, endobronchial ultrasound‐guided transbronchial needle aspiration (EBUS‐TBNA), and computed tomography (CT)‐guided biopsy from primary or metastatic sites. During the sampling with the TBLB, large biopsy forceps (large EBUS‐GS; FB‐231D; Olympus Medical Systems) were used at least five times. During the sampling using EBUS‐TBNA, the biopsies were performed at least three times, whenever possible.

### 2.4 Tumor specimen preparation

Tumor samples were fixed in 10% neutral‐buffered formalin solution for 6–24 h, embedded in paraffin wax, and subjected to histopathological examination. Histological diagnosis was performed by a pathologist according to the World Health Organization (WHO) lung tumor classification.^8^ After pathological diagnosis, molecular biomarker testing was performed for all NSCLC cases. We selected molecular biomarker testing based on two pathological factors that may affect the success rate of NGS analysis: tissue surface area and tumor content rate.^9^ If more than one tissue sample was available, then the sample with a larger tissue surface area and a higher tumor content rate were submitted for analysis. A tumor content rate of at least 30% is recommended for ODxTT‐M, and at least 20% for the AmoyDx PLC panel. When the tumor samples contained enough tissue surface area and tumor content rate, the number of glass slides submitted for the study was 10/20 glass slides with 5‐μm‐thick tissue strips attached. The submitted tumor volume was calculated as the product of tumor surface area, number of slides, and tissue thickness: tumor volume = tumor surface area (mm^2^) × number of slides (10 or 20 slides) × 5 μm per slide. In cases where it was difficult to secure the number of slides, the actual clinical examination was given priority and was excluded from this analysis. To equalize the number of specimens submitted, thin sections were prepared from tumor tissues for the ODxTT‐M and AmoyDx PLC panels, and the same number of slides was submitted for each. One set of samples was submitted to the SRL Laboratory (Tokyo, Japan), a commercial Japanese laboratory, at which ODxTT‐M was performed, and the other set was submitted to a laboratory at Riken Genesis (Tokyo, Japan), at which the AmoyDx PLC panel was performed.

### 2.5 DNA and RNA quantification and AmoyDx PLC panel analysis

DNA/RNA was extracted from the NSCLC tissue samples and purified using the Qiagen AllPrep DNA/RNA FFPE Kit at the Riken Genesis Laboratory. Following extraction, the optical densities (OD) of the DNA and RNA samples were measured using a NanoDrop 1000 spectrophotometer (Thermo Fisher Scientific). The DNA and RNA purity was determined from the absorbance ratio at 260/280 nm wavelength (A260/A280), and an A260/A280 value of 1.8–2.0 was used. In addition, DNA/RNA concentrations were quantified using a Qubit fluorescence assay (Thermo Fisher Scientific). The extracted DNA from the FFPE tissue concentration was adjusted to 1.5 ng/μL and the RNA concentration to 10 to 100 ng/μL. DNA and RNA multiplex PCR were used to amplify the target regions and detect somatic mutations. Simultaneously, hotspot mutations (single nucleotide variations, deletions, and insertions) and copy number variations were detected in the DNA‐based sequences, whereas fusion gene changes were detected in the RNA‐based sequences.

### 2.6 Companion diagnostic test (CDx)

All patients underwent genetic testing approved by medical insurance as a companion diagnosis. When a sufficient specimen volume was collected, the specimens were preferentially subjected to ODxTT‐M. In cases, where the SRL laboratory to which the specimen was submitted determined that the specimen volume was insufficient, the AmoyDx PLC panel was used. In cases where the specimen volume was deemed insufficient at the laboratory to submit multiple tests, priority was given to the Cobas® *EGFR* mutation test (cobas EGFR: Roche Molecular Systems) for *EGFR* mutation detection.

In this study, we defined analytical “success” as samples that were successfully reported to be positive or negative for nine driver mutations, including *EGFR*, *BRAF* V600E, *ALK, ROS1, KRAS, MET* exon 14 skipping, *RET, HER2*, and *NTRK*. If ODxTT‐M detected *MET* exon14 skipping, it was confirmed by ArcherMET companion diagnostic (Invitae Corp., San Francisco, CA, USA). If ArcherMET did not confirm the *MET* exon14 skipping, the ODxTT‐M test result was considered a false positive.

### 2.7 Statistical analysis

The analytical success and detection rates for the nine driver mutations in each test were analyzed for all enrolled patients. The analytical success and detection rates for the nine driver mutations achieved with ODxTT‐M and AmoyDx PLC panel were compared with Pearson's χ^2^ test. The discordance rates between the ODxTT‐M and AmoyDx PLC panels were analyzed in patients who successfully completed both analyses. Statistical significance was defined as a *p* < 0.05.

## 3 RESULTS

### 3.1 Patient characteristics

**FIGURE 1:**
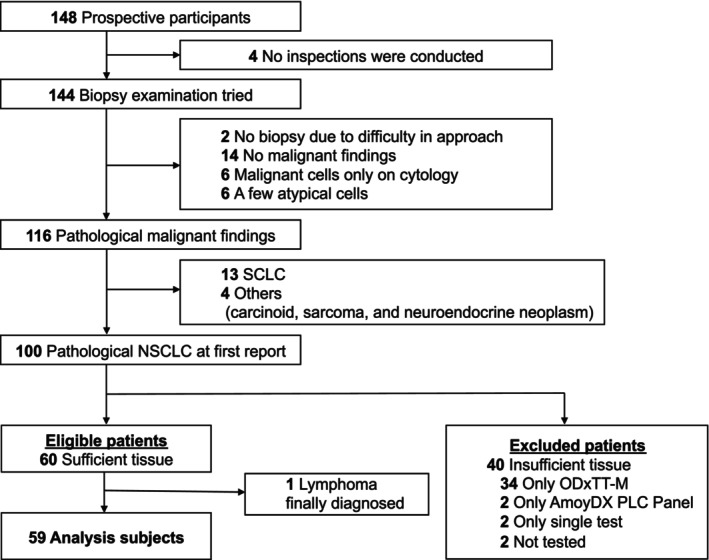
Consort diagram. AmoyDx PLC panel, AmoyDx Pan Lung Cancer PCR Panel; NSCLC, nonsmall‐cell lung cancer; ODxTT‐M, Oncomine Dx Target Test Multiple; SCLC, small‐cell lung cancer.

Of the 148 patients who provided consent, 144 underwent biopsy testing, and 116 exhibited malignant diagnosis. Apart from 13 SCLC and three other cases, 100 cases were pathologically diagnosed as NSCLC in the initial report. Of these, 60 patients with sufficient tissue volume were eligible for the study, and the specimens were sent to the Riken Genesis Laboratory. However, one patient was excluded because the final pathological diagnosis was lymphoma. Consequently, 59 NSCLC samples were subjected to simultaneous AmoyDx PLC panel and ODxTT‐M analysis (Figure 1).

The characteristics of the 59 specimens are summarized in Table 1. Tumor tissues were sampled by TBLB (*n* = 33), EBB (*n* = 2), EBUS‐TBNA (*n* = 21), or CT‐guided biopsy (*n* = 2). The mean tumor surface area was 12 mm^2^ (range 0.13–150). The mean tumor content rate was 47.4% (range, 1.5–92.5). Most patients were diagnosed with adenocarcinoma (*n* = 30), followed by SQ (*n* = 19), and “not otherwise specified” (NOS; *n* = 7). The number of glass slides submitted was 10 in 36 cases and 20 in 23 cases.

**TABLE 1:** Tumor specimen characteristics.

Characteristics	*n* = 59
Biopsy methods	
TBLB/EBB/EBUS‐TBNA/CT‐guided biopsy/lymph node biopsy	33/2/21/2/1
Tumor surface area (mm^2^), mean ± SD	12.0 ± 25.4
<1/1–2/2–4/4<	5/13/17/24
Tumor content rate (%), mean ± SD	47.4 ± 22.0
<20/20–30/30<	6/7/46
Histology	
Adeno/SQ/NOS/other	30/19/7/3
Number of glass slides submitted	
10/20	36/23

Abbreviations: CT, computed tomography; EBB, endobronchial biopsy; EBUS‐TBNA, endobronchial ultrasound‐guided transbronchial needle aspiration; NOS, not otherwise specified; SQ, squamous cell carcinoma; TBLB, transbronchial lung biopsy.

### 3.2 Test success rate and driver mutation frequency from each genetic analysis

**FIGURE 2:**
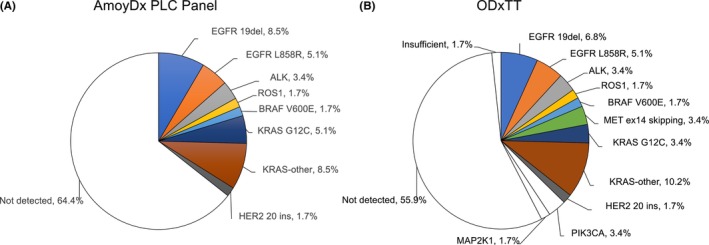
Frequency of detected mutations from AmoyDx Pan Lung Cancer PCR Panel (AmoyDx PLC panel) (A) and Oncomine Dx Target Test Multiple (ODxTT‐M) (B).

The AmoyDx PLC panel and ODxTT‐M analysis results are shown in Figure 2. The AmoyDx PLC panel successfully analyzed all cases, no failures occurred, and the test success rate was 100% (90% CI; 95.1% to 100%), which met the primary endpoint. The ODxTT‐M success rate was 98.3%, and only one sample had insufficient nucleic acid content. This one case was biopsied with EBUS‐TBNA and displayed a tumor surface area of 0.55 mm^2^ and a tumor content rate of 92.5%. The frequency of the nine driver mutations detected in each test was 35.9% for the AmoyDx PLC panel and 37.3% for ODxTT‐M across all patients (P = 0.848). Genetic alteration detection by the AmoyDx PLC panel was as follows: *EGFR* Ex.19 deletion (*n* = 5, 8.5%), *EGFR* L858R (*n* = 3, 5.1%), *ALK* fusion (*n* = 2, 3.4%), *ROS1* fusion (*n* = 1, 1.7%), *BRAF* V600E mutation (*n* = 1, 1.7%), *KRAS* G12C mutation (*n* = 3, 5.1%), *KRAS* other mutations (*n* = 5, 8.5%), and *HER2* Ex.20 insertion (*n* = 1, 1.7%). In addition to the nine driver mutations detected by ODxTT‐M, two gene mutations were detected in *PIK3CA* and one in *MAP2K1*. When limited to 30 adenocarcinoma cases, the frequency of the nine driver mutations detected was 56.7% (*n* = 17) with the AmoyDx PLC panel and 60% (*n* = 18) with ODxTT‐M.

### 3.3 Discordance between ODxTT‐M and AmoyDx PLC panel

Of the 58 cases in which both NGS and AmoyDx PLC panels were successful, discordant results were found in seven cases (12.1%) (Table 2). In case 1, the *EGFR* exon 19 variant did not mount in the ODxTT‐M test and was detected only by the AmoyDx PLC panel analysis. The *EGFR* exon 19 variant, which was not detected by ODxTT‐M, was confirmed to be L747P by the Oncomine Comprehensive Assay v3 (OCA v3; Thermo Fisher Scientific), which was performed as part of a lung cancer genomic screening project for individualized medicine in Japan (LC‐SCRUM) (UMIN ID: UMIN000010234). In cases 2–4, *MET* exon 14 skipping was detected in the ODxTT‐M but not in the AmoyDx PLC panel. In all cases, it was not detected by ArcherMET and was therefore considered a false positive by ODxTT‐M. The *MET* exon 14 skipping read counts in these patients (cases 2, 3, and 4) were 86, 111, and 46, respectively. In case 5, the *KRAS* G12F mutation identified in ODxTT‐M was determined to be a compound mutation of *KRAS* G12C and *KRAS*‐other in AmoyDx PLC panel. In case 6, *KRAS*‐other was detected only in the AmoyDx PLC panel. In contrast, in case 7, *KRAS* G13D was detected only in ODxTT‐M.

**TABLE 2:** Discordance cases between Oncomine Dx Target Test multiple (ODxTT‐M) and AmoyDx Pan Lung Cancer (PLC) Panel.

Case	Biopsy methods	Histology	Tumor surface area (mm^2^)	Tumor content rate (%)	AmoyDx PLC panel	ODxTT‐M	*MET* Copy Count	ArcherMET	OCA
1	TBNA	Adeno	0.7	10	*EGFR* exon19^a^	Not detected	–	–	*EGFR* L747P
2	TBNA	Adeno	9.15	50	Not detected	MET^a^	86	Not detected	–
3	EBB	Squamous	2.52	80	Not detected	MET^a^	111	Not detected	Not detected
4	TBLB	Adeno	0.2	55	EGFR 19del	EGFR 19del	–	Not detected	EGFR 19del
					Not detected	MET^a^	46	Not detected	Not detected
5	TBNA	Adeno	12.5	50	KRAS G12C + other	*KRAS* G12F	–	–	*KRAS* G12F
6	TBLB	Squamous	1.93	30	KRAS other	Not detected (VAF 3.2%)	–	–	–
7	TBNA	Adeno	7.1	52.5	Not detected	*KRAS* G13D			*KRAS* G13D

Abbreviations: AmoyDx PLC panel, AmoyDx Pan Lung Cancer PCR Panel; EBB, endobronchial biopsy; OCA, Oncomine Comprehensive Assay v3; ODxTT‐M, Oncomine Dx Target Test Multiple; TBLB, transbronchial lung biopsy; TBNA, transbronchial needle aspiration.

^a^ Diagnosis as a false call.

### 3.4 Nucleic acid concentration comparison detected by Qubit and Nanodrop

**FIGURE 3:**
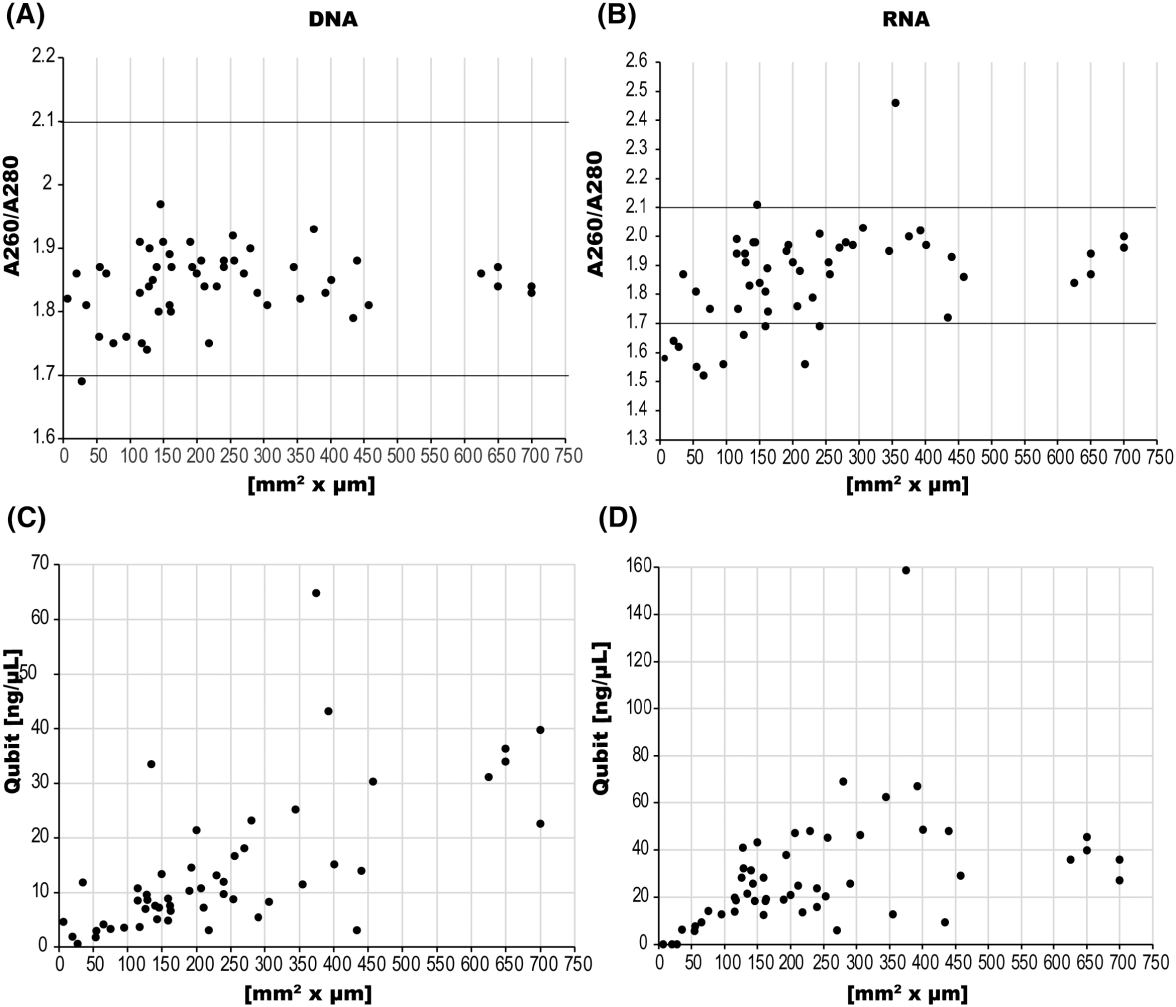
DNA (A) and RNA (B) Quality as per A260/A280 and the relationship between sample volume and DNA (C) and RNA (D) quantity by Qubit.

The relationship between DNA and RNA purity, determined from A260/A280, and the amount of tissue per specimen is shown in Figure 3A,B. The median values of A260/A280 for DNA and RNA were 1.86 and 1.91, respectively. The A260/A280 values were outside the recommended range in one case for DNA and 12 cases for RNA. For samples <50 × 10^−3^ mm^3^, the A260/A280 values for both DNA and RNA were often out of range. Both DNA and RNA concentrations measured by Qubit strongly correlated with tissue size (*R* ^2^ = 0.5382, *R* ^2^ = 0.6495) (Figure 3C,D). However, in three samples smaller than 50 × 10^−3^ mm^3^, the RNA concentration was below the Qubit measurement limit.

**FIGURE 4:**
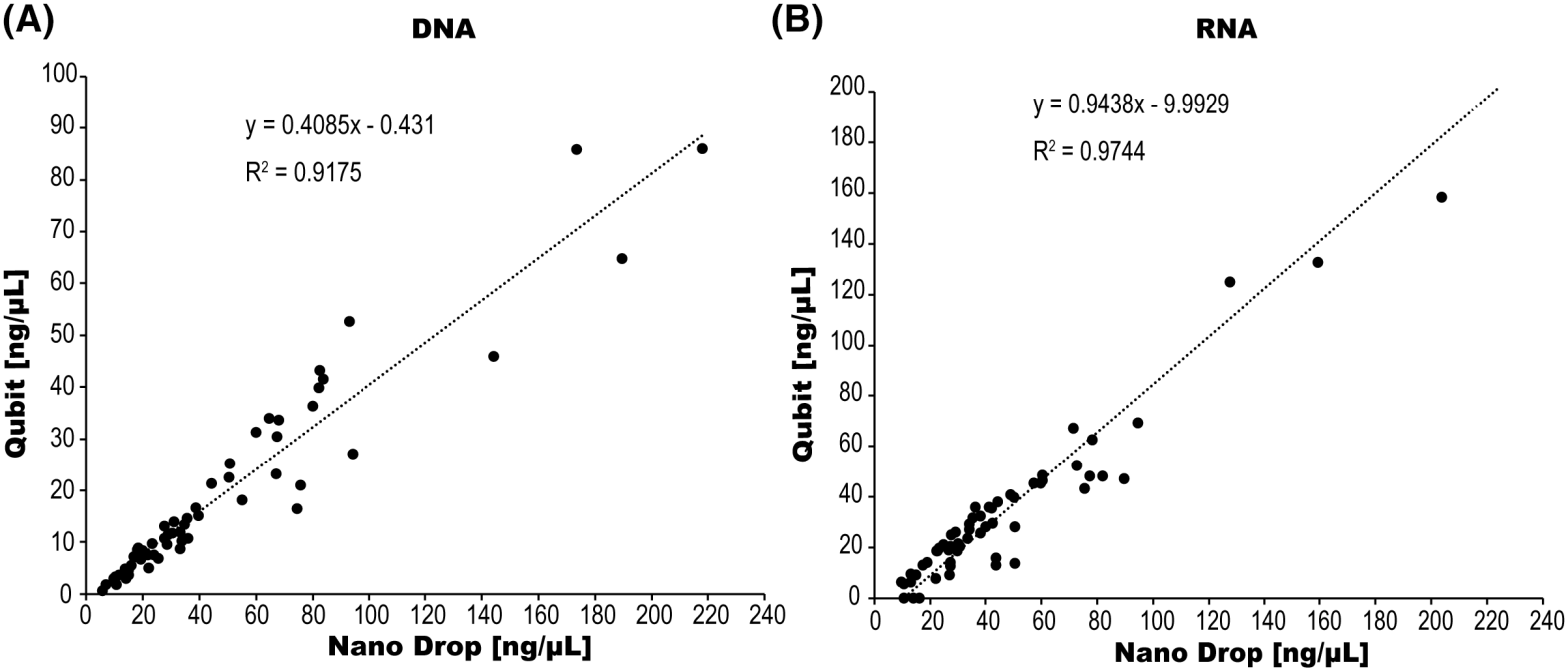
Concentration comparisons quantified by NanoDrop and Qubit in DNA (A) and RNA (B).

Concentration comparison quantified by NanoDrop and Qubit in DNA and RNA is shown in Figure 4A,B, respectively. The absolute DNA concentration values were higher when measured with NanoDrop than when measured with Qubit. A strong correlation was observed between the two methods (*R* ^2^ = 0.9175). In contrast, the RNA concentrations were similar when measured with the NanoDrop and Qubit, indicating a strong correlation between the two methods (*R* ^2^ = 0.9744).

## 4 DISCUSSION

This study compared the AmoyDx PLC panel to ODxTT‐M using biopsy specimens in detecting nine driver mutations essential for the treatment of patients with advanced NSCLC. The AmoyDx PLC panel showed a success rate of 100%, which was higher than that of ODxTT‐M. When comparing the AmoyDx PLC panel and ODxTT‐M, some detection rate differences were detected among the nine driver mutations. Much of this discrepancy is due to differences in the number of detectable variants and the sensitivity of each test method.

The AmoyDx PLC panel detected mutations in 35.9% of patients with NSCLC, with a 0% failure rate. ODxTT‐M failed in one case because of insufficient nucleic acid levels, whereas the AmoyDx PLC panel failed in none of the cases. This further proves that the AmoyDx PLC panel has a higher success rate than the ODxTT‐M panel. The mutation‐positive rate in this study was lower than that in previous reports, and this was mainly due to the patients' background; adenocarcinoma accounted for only approximately half of the patients in this study. Kunimasa et al. reported that the AmoyDx PLC panel detected mutations in 54.9% of cases with a failure rate of 1.5%.^10^ Of 406 patients included in this study, 81.3% had lung adenocarcinoma. Furthermore, their study also showed that the AmoyDx PLC panel had a higher analysis success rate than NGS, but the NGS method used in the study was not ODxTT‐M. We previously evaluated the performance of ODxTT‐M and the Cobas EGFR in detecting EGFR mutations. Similar to the current result, the success rate of PCR‐based Cobas EGFR was slightly higher than that of ODxTT.^7^ The analytical success rate was higher with PCR‐based methods, likely due to the simpler testing process compared with NGS‐based methods. This shortened the analysis time, enabling rapid analysis results.^10^

The detection rates of the nine driver mutations by the AmoyDx PLC panel and ODxTT‐M were similar; however, seven of the 59 cases showed discordance between the results of the two tests. These differences were mainly due to the differences in the sensitivity of the detection methods and the gene variants targeted in each test. Various *EGFR* mutation variants exist among these nine driver mutations. We also found 2.5% discrepancies between ODxTT‐M and Cobas EGFR in our previous study, and these discrepancies were mainly due to the difference in detectable *EGFR* variants.^7^ Frequent major mutations are covered by both the AmoyDx PLC panel and ODxTT‐M tests; however, for minor mutations, the variants and number of mutations that can be detected by each test differ. NGS analysis performed at LC‐SCRUM revealed a rare *EGFR* mutation (L747P) in exon 19. The AmoyDx PLC panel detects *EGFR* L747P as an *EGFR* del19 mutation, which is not listed as a detectable variant and is not intended to be detected. *EGFR* L747P is caused by a two‐base pair mutation (c.2239_2240TT>CC) at codon 747. The *EGFR* L747P mutation responsiveness to EGFR‐TKIs is unknown, but case reports indicate that lung adenocarcinoma patients with the *EGFR* L747P mutation were not sensitive to first‐ and third‐generation EGFR‐TKIs but were sensitive to second‐generation EGFR‐TKIs.^11^ Furthermore, afatinib, a second‐generation EGFR‐TKI, potently inhibits p.L747P mutant cells, reduces EGFR phosphorylation and downstream signaling, and significantly suppresses p.L747P mutant tumor growth.^11^ The p.L747P mutation may exacerbate the response to third‐generation EGFR‐TKIs compared to the classical *EGFR* exon 19 deletion. Therefore, it is concerning that PCR‐based testing identifies this specific mutation as an equivalent result to classical *EGFR* exon 19 deletion. NGS should be performed to detect this rare mutation and guide accurate TKI use in clinical settings. However, NGS‐based ODxTT‐M cannot detect this mutation, complicating identification of this mutation in clinical practice.

We observed *KRAS* discordance in three cases. *KRAS* G12C coexists with other activating *KRAS* mutations in NSCLC. Coexisting *KRAS* mutations were observed in 8% of *KRAS* c.34G>T mutant NSCLC tumors, with *KRAS* c.35G>T being the most frequently detected.^12^ Co‐occurrence of the c.34G>T and c.35G>T mutations in cis is converted to *KRAS* G12F, whereas in trans, both KRAS G12C and G12V proteins are generated; however, the AmoyDx PLC panel cannot differentiate between the two. Notably, *KRAS* G12F lung cancer models have reduced sensitivity to G12C‐specific inhibitors in vitro compared with *KRAS* G12C lung cancer cell lines.^12^ Therefore, the AmoyDx PLC panel results may be misleading for patients with *KRAS* G12F because these patients may be resistant to the KRAS inhibitor. The second case is that of *KRAS*‐other, which could be detected by the AmoyDx PLC panel but not by ODxTT‐M. This may be due to differences in the detection sensitivity. ODxTT‐M detected *KRAS* G12D at an allele frequency of 3.2%, which was below the positivity threshold. This result demonstrates the high sensitivity of PCR‐based testing. The third case was that of *KRAS* G13D, which could only be detected in ODxTT‐M; however, the AmoyDx PLC panel does not cover this mutation.

However, the issue with ODxTT‐M is that *MET* may be falsely detected. In a previous study of 26 samples that were positive for *MET* exon 14 skipping by ODxTT‐M, eight (30.8%) were not skipped by ArcherMET. The majority of discordant samples had read counts of <800 copies of the MET(13)‐MET(15) product by ODxTT‐M, which is considered a false positive for ODxTT‐M in samples with relatively low read counts. Here, three patients had discordant *MET* exon 14 skipping results, and all three cases had a low number of copies, which was considered a false positive by the ODxTT test.^13^ In this study, three cases had discordant *MET* exon 14 skipping results in the ODxTT‐M and AmoyDx PLC panels, and all three cases were considered false positives by the ODxTT‐M test because of the low copy number detected by ODxTT‐M and the fact that it was not detected by ArcherMET. Although a study with a small number of cases was conducted, the AmoyDx PLC panel results may escape false positives for *MET* exon 14 skipping, as well as ArcherMET. The fact that the AmoyDx PLC panel test does not require *MET* confirmation can shorten the diagnostic time, which is especially advantageous for patients with advanced NSCLC requiring urgent treatment.

The nucleic acid sample concentration and purity were measured using a NanoDrop in an AmoyDx PLC panel, and their concentrations were measured using a Qubit in ODxTT. The most notable difference between Qubit and NanoDrop was that Qubit had higher assay accuracy than NanoDrop. However, the Qubit has a measurement limit. Therefore, in the low‐concentration region, it may fall below the measurement sensitivity. In this study, RNA concentrations from three small tumor samples of 50 × 10^−3^ mm^3^ or less were below assay sensitivity. DNA concentrations tended to be higher when measured with the NanoDrop than with the Qubit, whereas the RNA concentrations were similar. The decrease in assay volume by Qubit indicates DNA quality degradation such as modification characteristics in formalin‐fixed paraffin‐embedded (FFPE) tissues.^14^ Consequently, Qubit is superior to the NanoDrop for DNA quantification in FFPE tissues. However, the Qubit does not quantify DNA fragmentation and does not always accurately assess PCR amplifiable DNA. Therefore, poor quality cannot be assessed at the time of DNA quantification and may become apparent after NGS analysis. In NanoDrop, sample purity is indicated by the ratio of absorbance at 260 to 280 nm, with a ratio of 1.8 generally considered indicative of pure DNA. A significantly lower ratio suggests the presence of contaminants. Therefore, Simbolo et al. recommended first assessing sample quality with NanoDrop, followed by sample concentration quantification using Qubit.^15^ However, the lower the concentration, the greater the variability in the value measured by the NanoDrop, which is expected to affect the A260/280 ratio. In fact, the cases in this study in which the A260/280 ratio measured by NanoDrop was outside the recommended range were found in small tumor specimens. For samples <50 × 10^−3^ mm^3^, accurately estimating the sample quantity and quality using NanoDrop may prove difficult.

Our study presents several limitations. First, the study included only patients from whom sufficient sample volumes were collected to avoid interference with routine practice; thus, the tests were performed in cases with relatively large sample sizes. Therefore, the performance of the AmoyDx PLC panel in cases with small sample sizes was not adequately evaluated. Second, the proportion of non‐squamous cancers was low, possibly owing to the selection bias described above. Consequently, the proportion of the nine driver mutations detected was lower than that previously reported. Therefore, comparison between the AmoyDx PLC panel and ODxTT for the nine driver mutation positivity rates was limited. Third, DNA and RNA quality were evaluated only by the A260/230 ratio; however, the evaluation of cases with A260/230 deviating from the recommended values was inadequate. It is possible that the low nucleic acid content affected the results; however, this problem could be resolved by measuring and clarifying the DIN and RIN values.

Despite these limitations, our finding that the AmoyDx PLC panel analysis can be successfully performed on samples that are difficult to analyze with ODxTT‐M of <1 mm^2^ is useful in clinical practice.^5^, ^6^ Although the analysis was performed on a small number of cases, the issues with each test were revealed. In the AmoyDx PLC panel, the nucleic acid content was measured using a Nanodrop. This method may result in inaccurate nucleic acid content when the sample volume is small, especially <50 × 10^−3^ mm^3^, which may cause errors in adjusting the nucleic acid concentration and ultimately affect the analysis results' accuracy.

The ODxTT‐M is an amplicon sequence‐based hotspot panel test that uses primers spanning portions of the coding region to amplify each target site. Therefore, both the AmoyDx PLC panel and ODxTT‐M can only detect mutations in the targeted mutation hotspots. Comprehensive genomic profile (CGP) testing using hybrid capture methods such as FoundationOne CDx and the NCC OncoPanel can detect mutations, amplifications, and homozygous deletions in all coding regions of target genes, along with target cancer gene rearrangements in each panel.^3^ Thus, CGP tests can detect rare mutations that cannot be detected using hotspot panel tests such as the AmoyDx PLC panel and ODxTT‐M.^16^, ^17^ Therefore, even if a companion diagnostic test does not detect actionable genetic mutations, additional CGP testing should be considered.

## 5 CONCLUSION

AmoyDx PLC panel is a PCR‐based multiplex diagnostic test with a high success rate. The frequency of genes targeted for treatment that could be detected by the AmoyDx PLC panel was comparable to that of the mutations detected by ODxTT‐M. No companion diagnostic test method is perfect, and clinicians should understand the performance and limitations of the respective methods and be aware of false‐positive and false‐negative results.

## AUTHOR CONTRIBUTIONS

**Shuji Murakami:** Conceptualization (lead); data curation (lead); formal analysis (lead); funding acquisition (lead); investigation (lead); methodology (supporting); project administration (lead); resources (lead); software (lead); supervision (lead); validation (lead); visualization (lead); writing – original draft (lead); writing – review and editing (lead). **Kanako Shinada:** Conceptualization (lead); data curation (lead); project administration (lead); writing – original draft (supporting); writing – review and editing (supporting). **Yuka Otsutsumi:** Methodology (lead). **Fumiko Komine:** Methodology (lead). **Yuan Yuan:** Methodology (lead). **Junko Nakamura:** Methodology (lead). **Seigo Katakura:** Data curation (lead); writing – review and editing (supporting). **Tetsuro Kondo:** Data curation (lead); writing – review and editing (supporting). **Terufumi Kato:** Data curation (lead); writing – review and editing (supporting). **Tomoyuki Yokose:** Conceptualization (lead); methodology (supporting); project administration (supporting); writing – original draft (supporting); writing – review and editing (supporting). **Haruhiro Saito:** Data curation (lead); writing – review and editing (supporting).

## FUNDING INFORMATION

The authors disclose the receipt of the following financial support for the research, authorship, and/or publication of this article: AmoyDx PLC panel analyses were conducted by Riken Genesis Co., Ltd., Tokyo, Japan.

## CONFLICT OF INTEREST STATEMENT

Shuji Murakami reports personal fees from AstraZeneca, Chugai Pharmaceutical, Boehringer Ingelheim, Taiho Pharmaceutical, Ono Pharmaceutical, and Riken Genesis. Terufumi Kato reports grants and personal fees from MSD, Novartis, Ono Pharmaceutical, Pfizer, and Taiho Pharmaceuticals, personal fees from Daiichi Sankyo, F. Hoffmann‐La Roche, Nippon Kayaku, Nitto Denko, Shionogi Pharmaceutical, Sumitomo Dainippon, and Takeda, and grants from Astellas, Kyorin, Kyowa Kirin, and Regeneron. Haruhiro Saito reports grants from Chugai Pharmaceutical and AstraZeneca, and personal fees from Ono Pharmaceutical, Nippon Boehringer Ingelheim, MSD, and Novartis Pharma. The other authors report no conflicts of interest.

## ETHICS STATEMENT

This study was conducted in accordance with the provisions of the Declaration of Helsinki and approved by the Ethics Committee of Kanagawa Cancer Center Hospital, Japan (2021EKI‐148).

## PATIENT CONSENT STATEMENT

Written informed consent was obtained from all patients.

## Supporting information


Table S1.

Table S2.

Table S3.

## Data Availability

All data in the present study are available via the corresponding author (S.M, murakami.0260j@kanagawa-pho.jp).
